# Evaluation of Magnetic Resonance Imaging Features to Distinguish Lipomas from Atypical Lipomatous Tumours in the Extremities and Back

**DOI:** 10.3390/diagnostics16162598

**Published:** 2026-08-17

**Authors:** Mohamed Hafizudin Abdullah Sani, Muhamad Zhafir Nor Asikin, Suraya Aziz, Juliana Fairuz Maktar, Emilia Rosniza Mohammed Rusli, Levin Kesu Belani, Nor Hazla Mohamed Haflah

**Affiliations:** 1 Department of Orthopaedics and Traumatology, Faculty of Medicine, Universiti Kebangsaan Malaysia, Jalan Yaacob Latiff, Kuala Lumpur 56000, Malaysia; budinsudin@gmail.com (M.H.A.S.); mzhafir.asikin@gmail.com (M.Z.N.A.); levinkb@gmail.com (L.K.B.); 2 Department of Radiology, Faculty of Medicine, Universiti Kebangsaan Malaysia, Jalan Yaacob Latiff, Kuala Lumpur 56000, Malaysia; drsuraya@hctm.ukm.edu.my (S.A.); julianafairuzmaktar@gmail.com (J.F.M.); emilia.rosniza@hctm.ukm.edu.my (E.R.M.R.)

**Keywords:** ALT, diagnosis, imaging features, lipomatous tumours, MRI

## Abstract

**Background/Objectives**: Lipoma and atypical lipomatous tumours (ALTs) are the most common soft tissue tumours. Accurate differentiation is clinically important because management differs substantially between these two entities. Magnetic resonance imaging (MRI) has been widely investigated as a complementary diagnostic tool to histopathology confirmation. Nevertheless, the diagnostic accuracy and reproducibility of MRI in routine clinical practice remain debated. **Methods**: This study aims to first evaluate MRI features that differentiate lipomas and ALTs, using histopathology as the reference standard, and second, to evaluate interobserver agreement for each MRI feature. A cross-sectional study was conducted on 12 patients (eight males, four females; median age 67, ranging 25–75 years) with biopsy-proven ALTs and 36 patients (15 males, 21 females; median age 58, ranging 28–80 years) with biopsy-proven lipomas who underwent surgery between 2020 and 2025. MRI features, including tumour location, compartment involvement, margin, signal characteristics, septa thickness, nodular components, contrast enhancement, and maximum tumour diameter, were independently assessed by three radiology specialists who were blinded to the histopathological diagnosis. Interobserver reliability for each MRI feature was evaluated using Cronbach’s alpha and intraclass correlation coefficients (ICCs). **Results**: Incidence of tumour size greater than 100 mm was significantly higher in the ALT group (91.7%) compared to the lipoma group (51.4%). Fifty-eight percent of ALTs had septa greater than 2 mm compared to only 11.8% of lipomas. Contrast enhancement involving more than one-third of tumour volume was also significantly more frequent in the ALT group (33.3%) compared to the lipoma group (2%). All ALTs were located below the deep fascia. Strong interobserver agreement was seen with tumour location (ICC = 0.933), size (ICC = 0.954), compartment involved (ICC = 0.902), and septa thickness (ICC = 0.82). **Conclusions**: Tumours below the deep fascia with a maximum diameter more than 10 cm, septal thickness more than 2 mm, and contrast enhancement involving more than one-third of tumour volume are useful features that can differentiate ALTs from lipoma.

## 1. Introduction

Lipomatous tumours are the most common soft tissue tumours found in the extremities [1]. The majority are lipomas or atypical lipomatous tumours (ALTs), together accounting for 40–45% of all lipomatous tumours [2]. The annual incidences of lipomas and ALTs are 2 per 1000 persons and 0.35 per 100,000 persons, respectively [3]. According to the 2020 World Health Organization classification, ALTs refer to well-differentiated lipomatous lesions located in the extremities, trunk, and abdominal wall where surgery is generally curative [2]. They are categorised as intermediate tumours with locally aggressive features such as recurrence or dedifferentiation, making their identification clinically significant [4].

The distinction between ALTs and lipomas is essential due to their different management strategies [5]. Appropriate preoperative identification of ALTs is important for guiding surgical planning [6,7] and preventing recurrence and dedifferentiation. In contrast, surgical excision is not necessary for all lipomas unless symptomatic [8,9]. Consequently, reliable diagnostic methods that can distinguish between these entities before definitive treatment are of considerable clinical importance.

Histopathological examination is the gold standard for differentiating the two [10]. ALTs generally present with more nuclear atypia, karyomegaly, fibrous proliferation, lipoblasts, and mitotic activity than lipomas [10]. Histopathological examination demonstrates a sensitivity of 93.8% and specificity of 82.3% in distinguishing ALTs from lipomas [10]. Still, biopsy is invasive and inadequate tissue sampling may limit the ability to obtain a diagnosis [11,12]. In addition, several studies have reported that biopsy sampling error and tumour heterogeneity may occasionally complicate histopathological interpretation, particularly in large lipomatous lesions [13,14]. These limitations have encouraged increasing interest in non-invasive imaging modalities that may assist clinicians in differentiating ALTs from lipomas, circumventing biopsy and proceeding directly to treatment of the tumour.

Magnetic resonance imaging has emerged as a non-invasive alternative due to its superior soft tissue contrast [15]. Imaging features such as tumour size, depth, septa thickness, fat content, and contrast uptake may distinguish ALTs from lipomas [16,17]. ALTs are typically larger and deep-seated, with thick irregular septa greater than 2 mm and lower fat content, whereas lipomas are smaller, superficial, and demonstrate homogeneity with surrounding subcutaneous fat [4]. Signal intensity in T1, T2, and fat-suppressed T1 MRI sequences typically favour lipomas, while incomplete fat suppression or septal enhancement suggest ALTs [12,18]. Overall, MRI demonstrates high diagnostic accuracy, with a reported sensitivity of 87.1% and specificity of 86.3% [12].

Nevertheless, the reliability of MRI in differentiating ALTs and lipomas is debated. Some studies argue that overlapping features limit MRI’s standalone diagnostic value [1,11]. Others support MRI and have proposed advanced diagnostic approaches based on imaging findings [19]. This study aims first to evaluate MRI features that differentiate lipomas and ALTs using histopathology as the reference standard, and second, to evaluate interobserver agreement for each MRI feature.

## 2. Materials and Methods

This was a hospital-based cross-sectional study conducted at Hospital Canselor Tuanku Muhriz, Kuala Lumpur, Malaysia, between March 2020 and March 2025. The study adhered to the Strengthening the Reporting of Observational Studies in Epidemiology (STROBE) guidelines for cross-sectional studies. Ethical approval was obtained from the UKM Research Ethical Committee.

All patients who underwent surgical excision of lipomatous tumours of the limbs and back during the study period and had preoperative magnetic resonance imaging (MRI) available for review were eligible. Patients were included if they had histopathological confirmation of either an ALT or lipoma. Cases without adequate imaging, incomplete clinical records, or indeterminate histopathological diagnosis were excluded. Sample size calculation was done using the formula for cross-sectional studies, utilising the reported ALT incidence of 13% by Mashima et al. 2020 [20]. Consecutive sampling was applied to minimise selection bias, with all eligible cases during the study period included. An additional allowance of approximately 15% was incorporated to account for potential incomplete records or inadequate imaging data in this retrospective review.

All MRI scans were performed following protocols from two different machines available in the institution, Siemens Magnetom Avanto 1.5T (Erlangen, Germany) and Siemens Verio 3T (Erlangen, Germany), with soft tissue tumour protocol MRI sequences including coronal STIR; T1 and T1 fat saturation post gadolinium; axial T1, T2, T2 fat saturation; and T1 fat saturation post gadolinium (Table 1).

All MRI scans were independently reviewed by 3 musculoskeletal radiologists, each with more than five years of experience. In cases of discrepant interpretations, the assessment of the most experienced reader was used as the final determination in subsequent analyses. All radiologists were blinded to the patients’ clinical information and final histopathological results. Images were reviewed using a dedicated DICOM viewing platform (Horos^®^, Annapolis, MD, USA).

The following tumour characteristics were assessed: tumour location, compartment involved, tumour size, maximum tumour diameter, tumour margin, signal characteristics, presence of contrast-enhancing areas, septal thickness, nodularity and diagnostic image quality.

Tumour location was determined according to the primary site of the tumour, categorised as upper limb, lower limb or trunk. The specific anatomical region was subsequently recorded. 

Compartment involvement was classified according to tumour location within the subcutaneous tissue, intermuscular compartment, intramuscular compartment or as multicompartmental when more than one compartment was involved.

Tumour size was measured in three orthogonal dimensions (anteroposterior × transverse × craniocaudal) using the imaging plane that best demonstrated the mass. The maximum tumour diameter was recorded and subsequently categorised as <100 mm or ≥100 mm.

Tumour margins were identified as either well-defined or pseudo-infiltrative. A well-defined margin was characterised by a discernible capsule or a clearly demarcated tumour boundary, whereas an ill-defined or irregular tumour interface with the surrounding tissue defined a pseudo-infiltrative margin.

Signal characteristics were assessed based on the estimated proportion of fat within the mass and categorised as predominantly fatty (75–100% fat), mixed (25–75% fat) and predominantly non-fatty (<25% or absence of visible fat).

The extent of contrast enhancement was categorised according to the proportion of the tumour demonstrating enhancement as either involving less than one-third (<1/3) of tumour size or at least one-third (≥1/3) of tumour size.

The presence of internal septation was assessed using a threshold of 2 mm. Septa ≤2 mm were considered thin, whereas those >2 mm were considered thick. The presence or absence of nodular components within the tumour was also recorded.

Diagnostic image quality was evaluated using a four-point Likert scale and categorised as excellent, good, moderate, or poor.

Assessments were recorded using standardised reporting forms, and interobserver reliability was evaluated using Cronbach’s alpha and intraclass correlation coefficients (ICCs). Statistical analysis was performed using IBM SPSS Statistics version 24 (IBM Corp., Armonk, NY, USA). Descriptive statistics were used to summarise the clinical, radiological, and surgical characteristics of patients with lipomatous tumours. Continuous variables were presented as medians and ranges, while categorical variables were expressed as frequencies and percentages.

Comparisons between atypical lipomatous tumours (ALTs) and lipomas were performed to identify clinical and radiological features associated with tumour type. As age was not normally distributed, differences in age between groups were analysed using the Mann–Whitney U test. Categorical variables, including gender, tumour location, anatomical region, surgical procedure, maximum tumour diameter, septal thickness, signal characteristics, contrast enhancement pattern, compartment involved, tumour margin, diagnostic image quality, and nodular component, were analysed using the Chi-square test or Fisher’s exact test where appropriate, based on expected cell frequencies.

## 3. Results

A total of 48 patients with lipomatous lesions were included in this study, comprising 12 patients with ALTs and 36 patients with lipomas. Clinical findings of patients with lipomatous tumours are summarised in Table 2. Although the median age of patients with ALTs was higher than that of the lipoma group, the difference was not statistically significant (*p* = 0.456). No significant association was found between gender and tumour type (*p* = 0.303).

Excision biopsy was performed in all patients with ALTs. In the lipoma group, all but one underwent excision biopsy. The remaining case of lipoma underwent wide local excision. The type of surgery performed did not differ significantly between groups (*p* = 0.446).

The radiological characteristics of patients with lipomatous tumours are presented in Table 3. We found four MRI features that were statistically significant in differentiating between ALTs and lipomas: maximum tumour diameter, septal thickness, contrast-enhancing area, and compartment involved. In the ALT group, 91.7% (11/12) had a maximum tumour diameter measuring more than 100 mm. In contrast, lipomas were distributed equally across both size categories.

**Figure 1:**
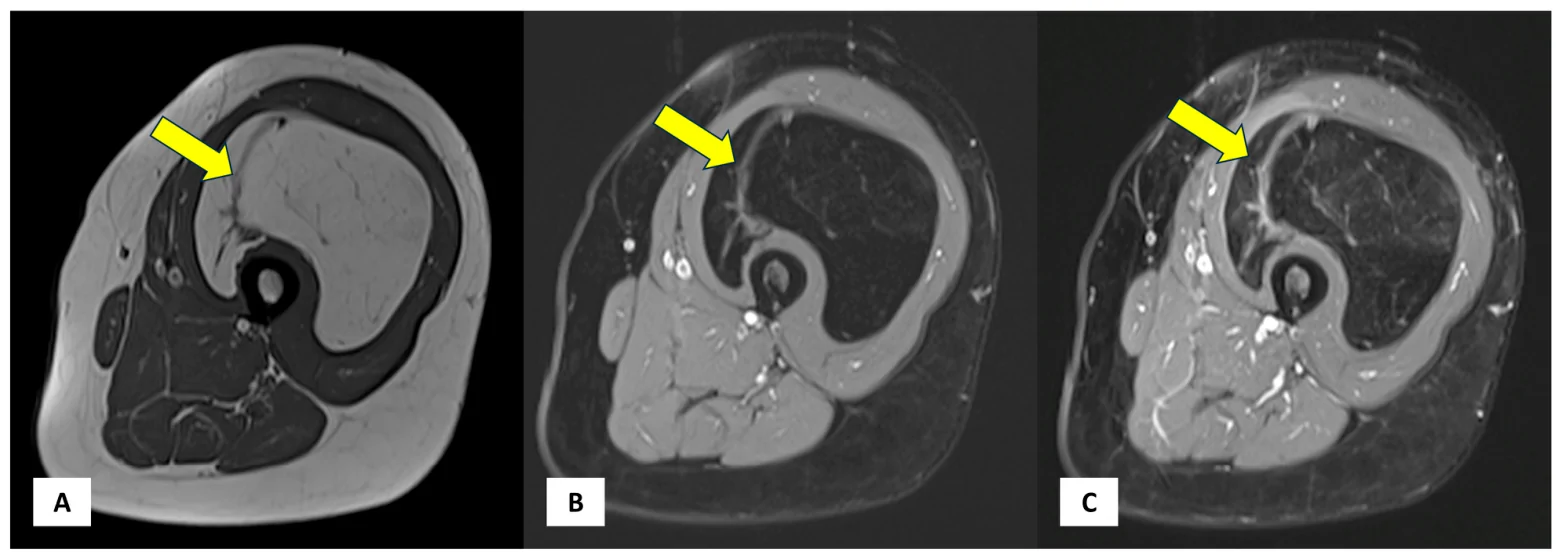
Axial magnetic resonance images of the left thigh demonstrate an intramuscular mass within the quadriceps muscle. (**A**) T1-weighted image reveals a predominantly hyperintense mass containing thick (>2 mm) hypointense septation (yellow arrow). (**B**) T1 fat-suppressed image shows marked signal suppression with minimal residual areas of high signal intensity. (**C**) Post-contrast T1 fat-suppressed image demonstrates heterogeneous enhancement of the internal septation and non-adipose components of the mass. The imaging features and histopathological examination are consistent with an atypical lipomatous tumour.

ALTs were associated with thick septa in 7 out of 12 cases (58.3%), whilst the majority of lipomas had thin septa (85%) (Figure 1). Thin septa were more common in lipomas (85.7%) than ALTs (14.3%).

**Figure 2:**
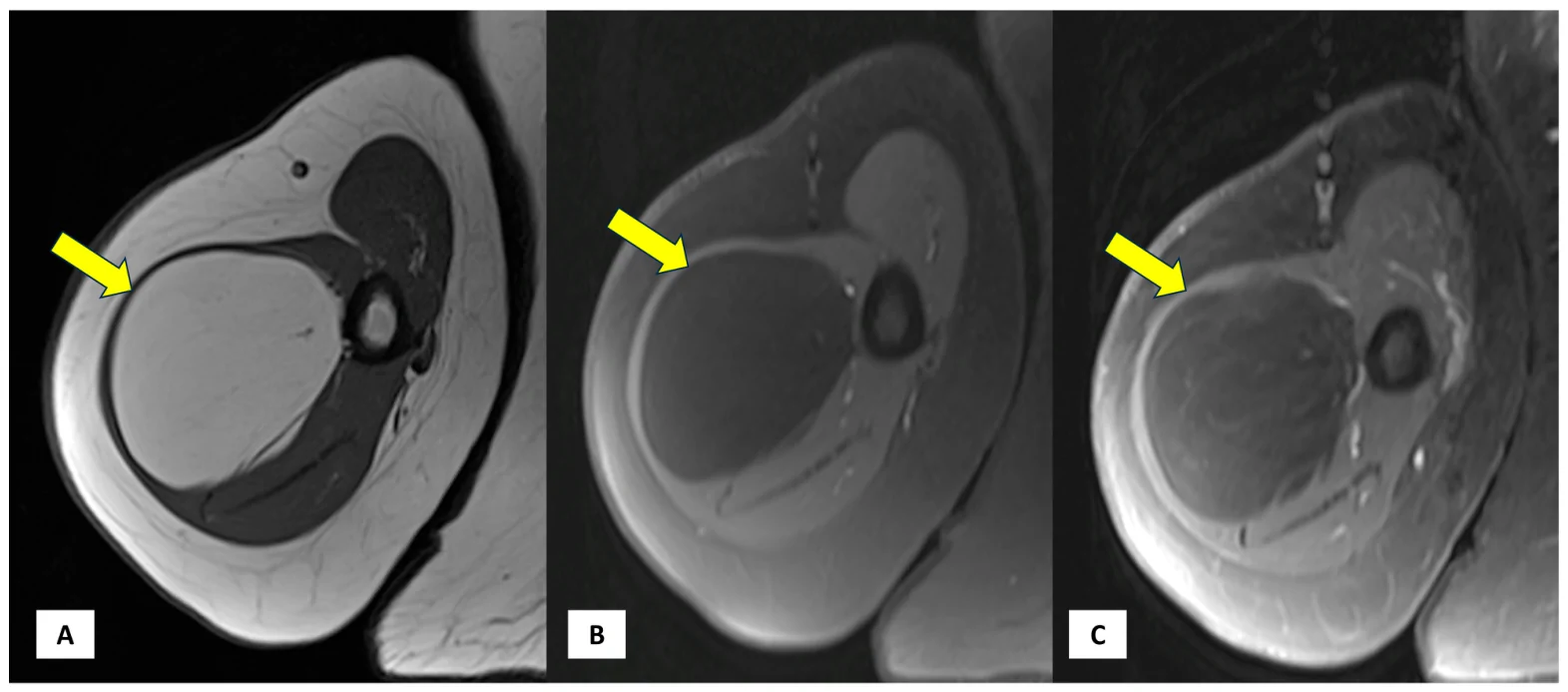
Axial magnetic resonance images of the right arm reveal a large intramuscular mass within the triceps muscle. (**A**) T1-weighted image shows a homogeneously hyperintense mass [yellow arrow]. (**B**) T1 fat-suppressed image demonstrates complete signal suppression, confirming the fatty nature of the mass. (**C**) Post-contrast T1 fat-suppressed image shows no appreciable internal enhancement. Imaging features and histopathological results are consistent with a benign intramuscular lipoma.

Contrast enhancement involving less than one-third of the tumour volume was more frequent in lipomas (81%) than in ALTs (19.0%) (Figure 2). Conversely, contrast enhancement involving more than one-third of the tumour volume was more frequent in ALTs (67%) than in lipomas (33%).

Concerning the location of the tumour, all ALTs were found below the deep fascia, either intramuscularly, intermuscularly, or both. Lesions in the subcutaneous region were all lipomas. However, lipomas were equally distributed between the superficial and deep layers.

The reliability of radiological assessments among the three radiologists is shown in Table 4. Parameters related to tumour location, size and compartment involved demonstrated very high interobserver agreement. Septa thickness showed moderate to strong reliability, suggesting acceptable agreement when distinguishing thin from thick septa. Tumour margin and signal characteristics showed the lowest interobserver agreement.

## 4. Discussion

Age, size, location, contrast enhancement and intensity have been identified as the most frequently used predictive variables across studies [21]. In our study, septa thickness, tumour size, contrast enhancement, and location demonstrated significant discriminatory value. When interpreted within the broader literature, these findings are consistent with established imaging characteristics of ALTs and support the generalisability of objective MRI markers in routine clinical practice.

Although overlap between large lipomas and ALTs was observed, ALTs consistently presented with larger tumour dimensions. In our series, all but one case of ALT had a diameter of more than 100 mm, with the widest measuring up to 34 cm × 14 cm × 8 cm. This observation parallels previous reports describing substantially greater tumour size and volume in ALTs [12,18,22]. Donners et al. identified a maximum tumour diameter of 11 cm as a single discriminator between an ALT and lipoma [22]. Knebel et al. reported that the probability of a lipomatous lesion being an ALT increased by 2.74 if the diameter exceeded 130 mm [17]. The deeper anatomical location of atypical lipomatous tumours (ALTs), typically beneath the deep fascia, may contribute to their larger size at presentation compared with lipomas. Similar to the findings reported by Knebel et al., all ALTs in our cohort were located deep to the subcutaneous tissues, potentially allowing continued growth before becoming clinically apparent. Nevertheless, consistent with previous studies, substantial overlap in tumour size was observed between ALTs and lipomas, particularly among larger lesions. These findings suggest that although tumour size may aid in differentiating ALTs from lipomas, it should not be considered in isolation and must be interpreted alongside other imaging characteristics. Consequently, although most ALTs are over 100 mm, large tumours are not necessarily indicative of ALT. Overlap is minimal in smaller tumours, and thus, we can conclude with some degree of confidence that tumours measuring less than 100 mm were more likely to be lipomas.

Septa thickness greater than 2 mm emerged as one of the most robust imaging markers. Thick septa were significantly more frequent in ALTs, consistent with prior studies identifying septal morphology as a central diagnostic discriminator [1,16,18]. Asano et al. incorporated septal thickness into a structured MRI scoring system with high reported diagnostic performance, whilst Scherrer et al. similarly demonstrated significantly greater septal thickness in ALTs compared with lipomas [4,12]. Moran et al. reported that septa ≥2 mm are suggestive of ALTs, but septa < 2 mm can be indicative of lipomas or ALTs. From our study, although more ALTs than lipomas had thick septa, a large proportion of ALTs also had thin septa. Hence, we can conclude that lesions with thin septa are suggestive of lipomas.

**Figure 3:**
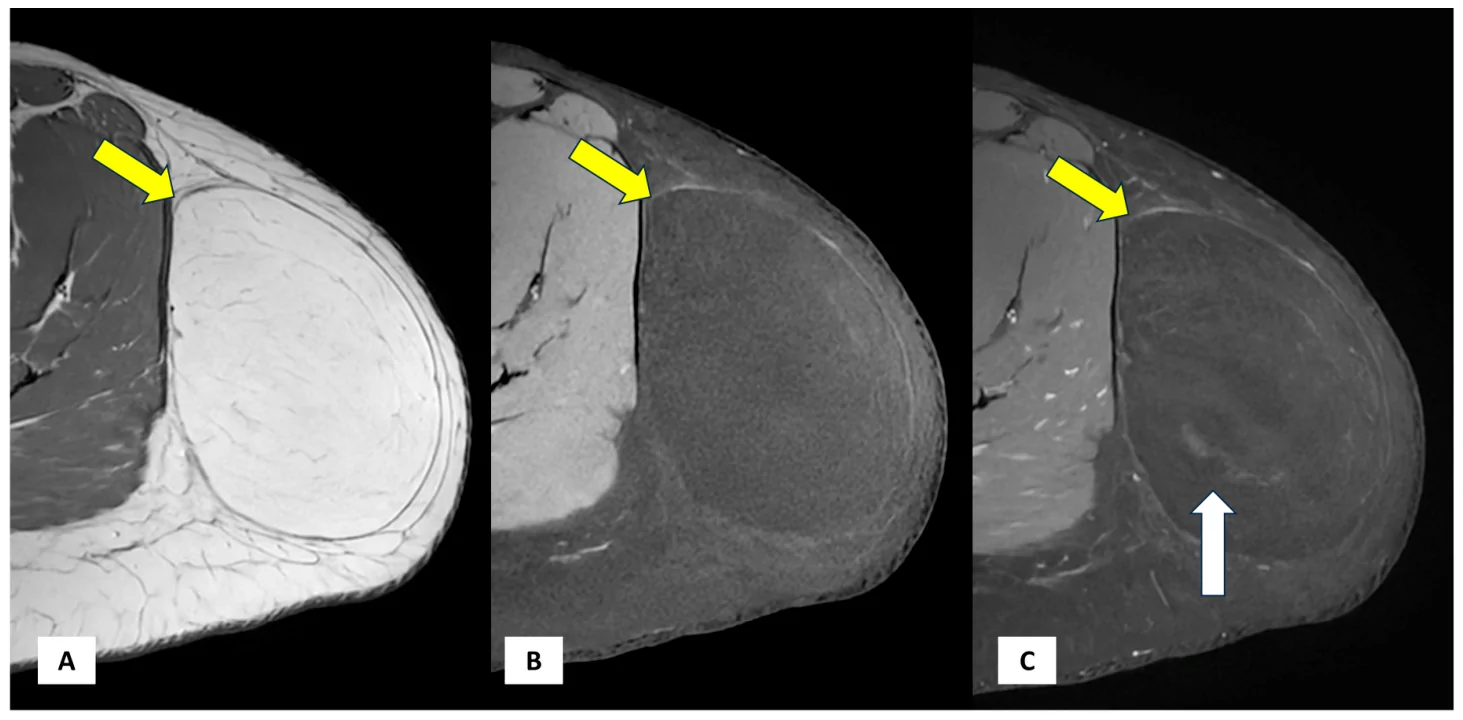
Axial magnetic resonance images demonstrate a large, encapsulated subcutaneous mass in the left gluteal region. (**A**) T1-weighted image shows a homogeneously hyperintense mass without appreciable septations, nodularity, or solid internal components [yellow arrow]. (**B**) T1 fat-suppressed image demonstrates complete signal suppression, confirming the fatty composition of the mass. (**C**) Post-contrast T1 fat-suppressed image reveals subtle focal nodular enhancement within the mass (white arrow), raising the possibility of an atypical lipomatous tumour. However, subsequent histopathological examination confirmed the diagnosis of a benign lipoma.

Contrast enhancement involving a substantial proportion of tumour volume was significantly associated with ALTs. This is concordant with the prior literature demonstrating that enhancement reflects fibrous septa and non-adipose cellular elements characteristic of ALTs [15,16]. Asano et al. highlighted enhancement patterns as integral to their diagnostic algorithm, and other series have similarly described heterogeneous enhancement as suspicious. In the current cohort, enhancement increased the likelihood of ALTs; however, reproducibility was lower than for quantitative parameters such as size and septal thickness (Figure 3). This finding suggests that while enhancement remains diagnostically meaningful, its application as a standardised criterion may benefit from clearer quantitative thresholds to enhance generalizability.

Signal characteristics also differed between ALTs and lipomas, with non-purely fatty or heterogeneous signal patterns observed in ALTs [15,18]. This pattern was not observed in our cohort. The interobserver agreement between the three radiologists was lowest for these MRI features which may explain why we found the result insignificant. Accordingly, signal heterogeneity should be considered a supportive feature rather than a primary standardised discriminator unless accompanied by more objective findings.

Moran et al. reported that 83% of lipomas in their series exhibited a well-defined margin whilst ALTs were associated with poorly defined and infiltrative margins [23]. We found no significant difference in our cohort, with the majority of lipomatous lesions showing a well-defined margin.

An important observation is the cumulative effect of indicative MRI features. Individually, septa thickness, tumour size, and enhancement extent increased the probability of ALTs to varying degrees. When multiple high-risk features coexisted, diagnostic likelihood increased substantially. Several structured scoring systems have been developed that demonstrate improved discrimination through composite criteria [1,4,11]. While formal multivariable modelling was not performed in this study due to a small sample size, the enrichment pattern observed supports the principle that integrated feature assessment enhances diagnostic stratification. The addition of clinical characteristics could further improve the prognostic value of MRI features. Scherrer et al., in their series, concluded that for tumours in the lower extremities with septation greater than 2 mm, the presence of a non-adipose mass, foci of high T2/STIR signal and contrast agent enhancement predicted a malignant lipomatous tumour with an accuracy of 0.941 (95% CI of 0.899–0.983; 87% sensitivity, 86% specificity) [12]. Weschenfelder et al. developed a scoring system using four clinical and three imaging features to distinguish lipoma from ALTs, achieving a sensitivity of 96.4% and a negative predictive value of 97.2% [24]. In our study, however, none of the clinical characteristics, including age and gender, showed any significant difference between ALTs and lipomas.

In a systematic review of 14 papers by Muhib et al., certain variables, despite their significance, showed only modest accuracy in predicting the final diagnosis when compared to MDM2 amplification detected via fluorescence in situ hybridisation (FISH) test [21]. Interobserver variability and differences in radiologists’ experience may account for this, further compounded by the qualitative nature of some of these variables. Interobserver variability can be minimised using simple MRI features rather than complex one [25]. Our study showed that moderate to high interobserver agreement was seen in parameters related to tumour location, size, compartment involved and septa thickness. Qualitative features such as signal characteristics showed the lowest interobserver agreement. Although the study utilised MR scanners of two different field strengths, (1.5 T and 3 T), the acquired images were of sufficient diagnostic quality for accurate interpretation. Since the evaluation did not target subtle signal abnormalities requiring high field strength, variation in field strength did not compromise the spatial resolution or lesion signal-intensity characteristics detected in this study.

Within contemporary diagnostic pathways, integration of MRI findings with molecular confirmation remains essential. Lipomas or ALTs can be predicted with high probability by evaluating the cytological findings of multinucleated cells, pleomorphism, and large, atypical cells in Pap-stained tissue specimens. MDM2 gene amplification testing via fluorescence in situ hybridisation (FISH) testing has been advocated for lipomatous cases with equivocal atypia, lipomatous lesions of the retroperitoneum/pelvis/abdomen, and deep-extremity lesions >10 cm in patients over 50 years of age [25,26]. However, molecular testing introduces additional costs and resource utilisation [27].

Some studies have suggested that when MRI demonstrates multiple characteristic features of an ALT, such as large tumour size, thick septa, and significant enhancement, the probability of an ALT is sufficiently high to guide clinical decision-making without routine molecular confirmation in all cases [4,27,28]. Objective MRI criteria guide biopsy selection and molecular testing rather than replace them. Nevertheless, MRI-guided approaches may be particularly relevant in resource-limited settings, where selective use of molecular testing could reduce diagnostic cost while maintaining clinical safety [27]. In our study, subcutaneous lesions measuring less than 100 mm, with thin septa and a contrast-enhancing area of less than 1/3 of the volume, were most likely lipomas, and in some cases biopsies were not performed.

The risk of dedifferentiation of liposarcoma from ALTs is approximately 10% of cases; hence, differentiating lipomas from ALTs is important [29]. In our study, we did not include liposarcoma since there is less radiological overlap between liposarcoma and its benign counterpart. Surgery is the mainstay treatment for all ALTs, whilst lipomas may be monitored and only require surgery if symptomatic [6,7,8,9]. Although marginal excision is adequate for ALTs, complete tumour excision must be achieved due to a higher recurrence rate in incomplete excision [6,7]. Wide local excision is unnecessary because of its higher associated morbidity, especially when the tumour is in close proximity to vital structures. Furthermore, any recurrence in the limbs and back can be monitored easily. One patient with a lipoma underwent wide local excision. The initial MRI interpretation by a general radiologist suggested features concerning for liposarcoma. Although this assessment did not align with our clinical impression, we elected to proceed with wide local excision to ensure oncological safety. In addition, the lesion was located within the subcutaneous tissue, allowing excision with adequate margins without significant additional morbidity.

This study is limited by a modest sample size and incomplete imaging data in a subset of cases. We acknowledge this as an important limitation since it will reduce statistical power and affect the precision of performance estimates. Nonetheless, the concordance of key imaging markers with the established literature strengthens confidence in their diagnostic relevance. Other limitations include discrepant interpretations that were resolved using the assessment of the most experienced radiologist rather than by consensus review, which may have introduced observer-related bias. Future multicentre studies incorporating multivariable modelling and standardised MRI scoring systems may further refine non-invasive differentiation strategies and enhance external validity.

## 5. Conclusions

Radiological assessment plays an important role in distinguishing atypical lipomatous tumours from lipomas. We found several MRI features that would aid the management of lipomatous tumours specifically influencing our decision regarding the necessity of biopsy. Due to the presence of some overlap of these MRI features between ALTs and lipomas, histopathological examination may be required in equivocal or borderline cases to dictate the course of future management.

## Figures and Tables

**Table 1:** MRI lower limb protocol for soft tissue tumours.

MRI Sequence	TR (ms)	TE (ms)	Slice Thickness (mm)	FOV (cm)	Matrix
Coronal and axial T1-weighted (T1W)	400	20	3	16–40	256 × 192
Coronal STIR	5000	50	3	40	256 × 192
Axial/coronal/sagittal post contrast T1 fat saturated (T1FS)	400	20	3	16–40	256 × 192
Axial T2 fat saturated (T2FS)	4000	100	3	16–24	320 × 224
Axial T2-weighted (T2W)	4000	100	3	16–24	256 × 192

**Table 2:** Clinical characteristics among patients with lipomatous tumours.

Variables	Group		*p*-Value
	ALT *N* = 12	Lipoma *N* = 36	
Age (Median; Range)	67; 25–75	58; 28–80	0.456
Gender			0.303
Male	8	15	
Female	4	21	
Location of Tumour			0.068
Upper Limb	3	13	
Lower Limb	9	20	
Back	0	3	
Anatomic Region			0.549
Thigh	7	10	
Arm	2	7	
Shoulder	0	7	
Buttock	2	3	
Back	0	3	
Forearm	1	1	
Other Body Parts	0	5	

**Table 3:** Radiological characteristics among patients with lipomatous tumours.

Variables	Group		*p*-Value
	ALT *N* = 12 (%)	Lipoma *N* = 36 (%)	
Maximum Tumour Diameter (mm)			0.005
<100	1 (5.3)	18 (94.7)	
>100	11 (38.0)	18 (62.0)	
Septa Thickness			0.001
Thin (≤2)	5 (14.3)	30 (85.7)	
Thick (>2)	7 (58.3)	5 (41.7)	
Signal Characteristics			0.023
Predominantly fatty	10 (21.8)	36 (78.2)	
Predominantly non-lipomatous	1 (100)	0 (0.0)	
Mixed	1 (100)	0 (0.0)	
Contrast Enhancing Area			0.005
Less than 1/3 of volume	8 (19.0)	34 (81.0)	
More than 1/3 of volume	4 (66.7)	2 (33.3)	
Compartment Involved			0.005
Subcutaneous	0 (0.0)	21 (100)	
Intermuscular/Intramuscular	12 (44.4)	15 (55.6)	
Tumour Margin			0.322
Well-defined	11 (23.9)	35 (76.1)	
Pseudo-infiltrative	1 (50.0)	1 (50.0)	
Diagnostic Image Quality			0.857
Excellent	11 (26.8)	30 (73.2)	
Good	1 (16.7)	5 (83.3)	
Moderate	0 (0.0)	1 (100)	
Poor	0 (0.0)	0 (0.0)	
Nodular Component			0.166
Absent	9 (22.0)	32 (78.0)	
Present	3 (42.9)	4 (57.1)	

**Table 4:** Reliability of radiological assessments among 3 radiology specialists.

Parameters	Intraclass Correlation Coefficient
Location of tumour	0.993
Compartment involved	0.902
Tumour margin	0.457
Signal characteristics	0.370
Contrast enhancing area	0.448
Septa thickness	0.802
Nodular component	0.661
Maximum tumour diameter	0.954
Diagnostic image quality	0.566

No recurrences were reported in either group.

## Data Availability

Research data are stored in an institutional repository and will be shared upon request to the corresponding author.
